# “Pathomorphogenic” Changes Caused by Citrus Bark Cracking Viroid and Transcription Factor TFIIIA-7ZF Variants Support Viroid Propagation in Tobacco

**DOI:** 10.3390/ijms24097790

**Published:** 2023-04-24

**Authors:** Jaroslav Matoušek, Kevin P. Wüsthoff, Gerhard Steger

**Affiliations:** 1 Biology Centre of the Czech Academy of Sciences, Institute of Plant Molecular Biology, Branišovská 31, 37005 České Budějovice, Czech Republic; matjj@seznam.cz; 2 Institut für Pysikalische Biologie, Heinrich Heine University Düsseldorf, D-40204 Düsseldorf, Germany; kevin.wuesthoff@hhu.de

**Keywords:** regulation of plant morphogenesis, viroid pathogenesis, transcription factors, transcriptome profiling, plant transformation, *Nicotiana tabacum*

## Abstract

Viroids are small, non-coding, pathogenic RNAs with the ability to disturb plant developmental processes. This dysregulation redirects the morphogenesis of plant organs, significantly impairing their functionality. Citrus bark cracking viroid (CBCVd) causes detrimental developmental distortions in infected hops (*Humulus lupulus*) and causes significant economic losses. CBCVd can infect cells and tissues of the model plant tobacco (*Nicotiana tabacum*), provided it is delivered via transgenesis. The levels of CBCVd in tobacco were enhanced in plant hybrids expressing CBCVd cDNAs and either the tobacco or hop variant of TFIIIA-7ZF, a viroid-mediated splicing derivative of transcription factor IIIA, which is important for viroid replication by DNA-dependent RNA polymerase II. The TFIIIA-7ZF variants can change the tobacco morphogenesis if expressed in leaves and shoots. In addition to the splitting of shoots, the “pathomorphogenic” network in hybrid plants expressing CBCVd and *Hl*TFIIIA-7ZF induced leaf fusions and malformations. Moreover, CBCVd can dramatically change another morphogenesis into teratomic and petal-like tissues if propagated above some limit in young transgenic tobacco microspores and anthers. By comparative RNA profiling of transgenic tobacco shoots bearing TFIIIA-7ZFs and CBCVd-transformed/infected anthers, we found a differential expression of many genes at *p* < 0.05. As the main common factor showing the differential up-regulation in shoot and anther tissues, a LITTLE ZIPPER 2-like transcription factor was found. We propose that this factor, which can interact as a competitive inhibitor of the also dysregulated homeobox-leucin zipper family protein (HD-ZIPIII) in apical meristem, is essential for a network responsible for some morphological changes and modifications of plant degradome within shoot meristem regulation and secondary xylem differentiation.

## 1. Introduction

To date, more than 25 plant diseases in more than 15 crops, including vegetables, fruit trees, and flowers, have been reported for viroids represented by small, circular, non-coding pathogenic RNAs, ranging from 246 to 434 nucleotides [1]. Viroid-host interactions induce more or less pronounced symptoms of disease; in some cases, latent infections without morphological symptoms were observed (for reviews, see e.g., [1,2,3]). Some morphological symptoms change the plant habitus, including dwarfing, leaf and fruit size, shape, and coloration, but in some cases the symptoms could be designated as “pathomorphogenic” when affecting the functionality of plant shoots, roots, organs such as leaves and flowers, or fruits, causing sterility or impairing plant propagation [4,5,6]. Citrus bark cracking viroid (CBCVd), which belongs to the *Pospiviroidae* family, is of particular interest; this disease is rather latent in citruses, but a variant adapted to hops (*Humulus lupulus* L.) causes bark cracking of the hop vine, extensive plant weakness, and retarded growth of shoots and roots. These degenerative disorders in hops are accompanied by complex metabolical changes, a depression of metabolomes, and disorders on the molecular level [7,8,9,10,11,12]. CBCVd has a wide host range and can be replicated in some *Solanacenaceae* species [9], including *N. tabacum*, where it can replicate but cannot move; that is, its propagation can be mediated only through plant transformations using infectious plant vectors [13] and its infection is, in general, without morphological symptoms. Recent results and our previous study [14] also suggest that CBCVd probably adopted, as other nuclear-replicating viroids, a splicing variant of the transcription factor TFIIIA, TFIIIA-7ZF with only seven zinc fingers, for their own replication by DNA-dependent polymerase II [15,16].

In a previous study [14], we integrated a splicing variant of the transcription factor IIIA from PSTVd-infected *N. benthamiana* (*Nb*TFIIIA-7ZF) into a plant vector (Figure S1A), transformed tobacco (*N. tabacum*), and demonstrated that *Nb*TFIIIA-7ZF, if overexpressed ectopically in tobacco, caused some unusual morphological changes [14]. In addition, the TFIIIA-7ZF transgene led to a depression of the plant “degradome” in young non-senescent plants, which was similar to viroid-restricting degradome in pollen [13,14]; therefore, we assumed a possible involvement of TFIIIA-7ZF in the physiological processes connected to viroid propagation, pathogenesis, or plant morphogenesis. Our previous analyses also demonstrated that the level of *Nt*TFIIIA-7ZF increased upon the viroid infection of tobacco (*N. tabacum*) and showed that the degradation complex undergoes some depression in young plants infected with hop viroids, such as apple fruit crinkle viroid (AFCVd) and CBCVd [14]. At the same time, these viroids propagate with higher integrity and levels in transgenic *N. tabacum* [14] and also in transgenic *N. benthamiana* [17].

In the present study, we analyzed viroid “pathomorphogenesis” using single transgenotes and crossing the combined transgenotes of TFIIIA-7ZF and CBCVd. In addition, we used gene profiling to analyze “pathomorphogenic” imbalancing of gene expression caused by TFIIIA-7ZF overexpression in tobacco shoots and due to the “forcing” of CBCVd propagation in the transformed tobacco male germline. We connected networks potentially relevant to viroid-modified TFIIIA, causing unusual morphological changes independent of the viroid, as well as more complex “pathomorphogenic” changes initiated over the threshold of restrictive CBCVd infection in tobacco anthers.

## 2. Results and Discussion

### 2.1. Splicing Variants of Viroid-Modulated TFIIIA: TFIIIA-7ZF from *N. benthamiana* and *Humulus lupulus* Support CBCVd Propagation in Transgenic Tobacco

The hop isolate of CBCVd (AC KM211547), analyzed in this study, causes particular morphogenic changes in hops, especially bark cracking, depression of rooting, and hops growing potential. The symptoms’ expressions are obviously caused by part of a CBCVd-induced pathogenesis network [8,10,11,12]. The modification of TFIIIA factor(s) in hops obviously play a significant role in the propagation of hop viroids, as indicated for HLVd by Patzak et al. [18]. While CBCVd causes severe pathogenesis in hops, there are negligible symptoms caused by CBCVd infection in *N. tabacum* and the infection proceeds rather in a latent form, as judged by visible symptoms in somatic tissues. Moreover, CBCVd can accumulate only in tissues transformed using infectious plant vectors because *N. tabacum* is not a natural host of CBCVd. We prepared such vectors bearing dimeric CBCVd driven by either 35S or pollen-specific promoters (Figure S2) during our previous studies [13,14]. CBCVd infection induced in transformed/infected tissues of tobacco is beneficial for the combinatorial analysis of “controlled” viroid infection with ectopically over-expressed *N. benthamiana*-specific and hop-specific TFIIIA-7ZF, which can be achieved by the formation of hybrid tobacco plants through the crossings of transgenotes bearing CBCVd and TFIIIA-7ZF transgenes. In the present work, we analyzed tobacco transformed with CBCVd and TFIIIA-ZF transgenes from *N. benthamiana*, hops, and corresponding hybrids to analyze their impact on viroid levels and induced morphogenic changes.

A sequence comparison of *Nb*TFIIIA-7ZF and *Hl*TFIIIA-7ZF revealed 60% identity on the amino acid (aa) level and 75% homology (Figure S4). Besides some deletions on the DNA level, there are less pronounced regions of homology of these proteins in their first third of the sequence and in the C-terminal parts (Figure S4). A comparison of the protein domains of *Nb*TFIIIA-7ZF and *Hl*TFIIIA-7ZF, using InterProScan, identified seven C2H2 zinc fingers in each of them, as expected (Figure S5). The domains were somewhat shifted relative to N termini, but seemed to be similarly distributed along the aa sequences. *Nb*TFIIIA-7ZF and *Hl*TFIIIA-7ZF were cloned into vector pJM14 (Figure S1), which was constructed earlier [13], and transformed into *N. tabacum*.

**Figure 1:**
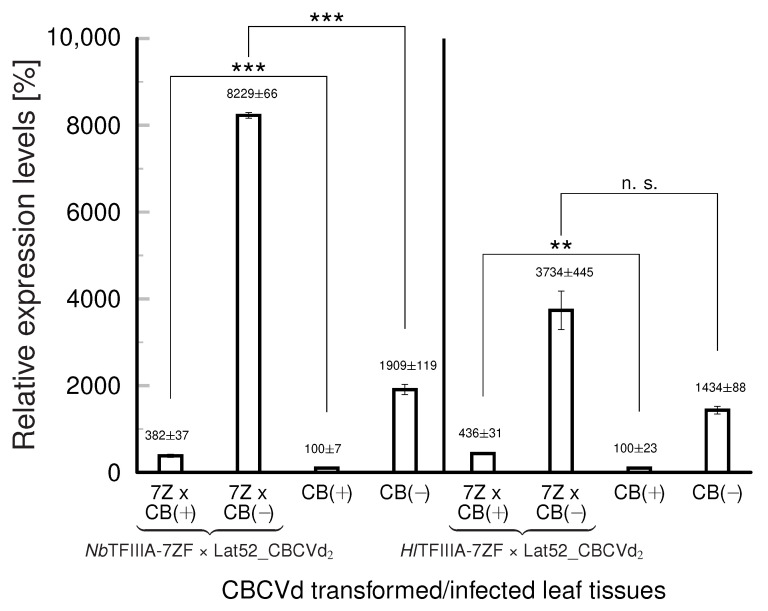
Quantification of (+) and (−) strands in CBCVd-transformed/infected tobacco. (**A**) Comparison of viroid levels in Lat52_CBCVd_2_ and *Nb*TFIIIA-7ZF × Lat52_CBCVd_2_ hybrids. (**B**) Comparison of levels in Lat52_CBCVd_2_ and *Hl*TFIIIA-7ZF × Lat52_CBCVd_2_ hybrid plants. Samples were collected after 40 days of cultivation from the upper third of the shoots. RNA was isolated and strand-specific qPCR was performed, as described in Materials and Methods. The levels of CBCVd (+) strands in Lat52_CBCVd_2_ leaves were taken as 100%. Relative expression levels were normalized to 7SL RNA. Columns represent the mean ± S.D. of two replicates of each PCR reaction. Statistically evaluated differences are shown by columns connected by lines (n.s., statistically non-significant differences at *p* < 0.1; **, statistically significant differences at *p* < 0.05; ***, statistically significant differences at *p* < 0.01).

To compare the possible impacts of TFIIIA-7ZF variants on viroid levels, crossings of TFIIIA-7ZF plants with Lat52_CBCVd_2_ transgenotes were performed (see Section 3 and Figure S2). The CBCVd levels were analyzed in the upper leaves of these hybrid plants 40 dpp using strand-specific RT-qPCR (Figure 1) and were compared to the levels in the Lat52_CBCVd_2_ transgenotes. The levels of CBCVd (+) and (−) strands were higher in both tobacco hybrids than in the single Lat52_CBCVd_2_ transformants. In addition, (−) strands prevailed over (+) strands; this is a specific characteristic of this viroid in *Nicotiana* plants and also in hops [9]. The propagation of CBCVd in *Nb*TFIIIA-7ZF × Lat52_CBCVd_2_ was stronger than in *Hl*TFIIIA-7ZF × Lat52_CBCVd_2_ when compared to Lat52_CBCVd_2_. In this case, the differences were significant for both (+) and (−) CBCVd strands, while in the case of *Hl*TFIIIA-7ZF, the difference was non-significant for (−) strands, but significant for (+) strands. These results are consistent with our previous finding [14] that tobacco transformed by the CBCVd expressed elevated level of *Nt*TFIIIA-7ZF and was more viroid. Here, we clearly demonstrated that ectopically over-expressed *Nb*TFIIIA-7ZF supports CBCVd replication in transgenic tobacco. This was the case also for *Hl*TFIIIA-7ZF, where enhanced levels of (+) strands were statistically significant. We cannot exclude from our results using hybrid tobaccos that, besides the introduced TFIIIA-7ZF transgenes, there is some contribution of *Nt*TFIIIA-7ZF to viroid accumulation.

A more detailed analysis was performed with *Hl*TFIIIA-7ZF × Lat52_CBCVd_2_ transgenic hybrids. In these plants, a lower level of degradome including *Nt*Tudor S-like nuclease *Nt*DCL, *Nt*AGO5, and pollen extracellular nuclease I were detected in tobacco leaves collected from the upper third of plant shoots 40 days after hybrid seedlings pricking (dpp) (Figure S6). This suggests that the *Hl*TFIIIA-7ZF transgene influenced the levels of these factors. The differences were significant although not as strong as previously detected for the *N. benthamiana* homologue *Nt*TFIIIA-7ZF [14]. Hybrid plants expressed a level of Neomycin phosphotransferase II transgene (NptII, resistance to Kanamycin) similar to the level in Lat52_CBCVd_2_ transformants; the difference was not statistically significant. The expression of hop-specific *Hl*TFIIIA-7ZF transgene was well-detectable in the hybrid but not in the Lat52_CBCVd_2_ plants (Figure S6).

### 2.2. Specific Morphogenic Changes Caused by Elevated Levels of TFIIIA-7ZF and CBCVd

**Figure 2:**
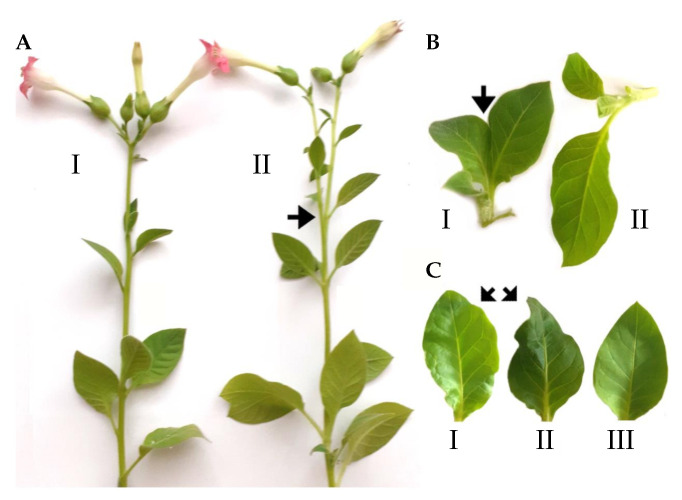
Some morphological deviations of *N. tabacum* hybrids *Hl*TFIIIA-7ZF × Lat52_CBCVd_2_ prepared using crossing between plant lines transformed with infectious dimers of CBCVd (Figure S1) and *Hl*TFIIIA-7ZF (Figure S5B). (**A**) Splitting of apexes causing branching of the main plant shoot as indicated by the arrow in II versus control I. (**B**) Fusion of leaf blades as shown by the arrow (I) compared to control II. (**C**) Leaf malformations in I and II versus control III.

In our previous work we found that *Nb*TFIIIA-7ZF caused numerous morphological deviations in transgenic tobacco, such as plant stunting, splitting of leaf petioles, pistils or apexes, irregular branching of shoots, formation of double-blade leaves, deformation of main shoots, and modification of glandular trichomes. In addition, there were some physiological symptoms such as a delay of aging in comparison to the untransformed controls [14]. Surprisingly, in the case of single transformants with *Hl*TFIIIA-7ZF, only one dominant macroscopic morphological deviation was observed: irregular branching of the main stem which split this tobacco shoot into two equal branches (Figure 2A, compare I and II) or three branches in a single lower position of the shoot [19]. In *Hl*TFIIIA-7ZF × Lat52_CBCVd_2_, we observed additional morphological deviations such as the fusion of leaf petioles and leaf blades (Figure 2B, compare I and II) and leaf blade bending and deformations (Figure 2C, compare I and II with standard leaf III). In thecase of *Hl*TFIIIA-7ZF transgene and even in hybrids, we did not observe significant stunting, fusion of single blades, deformation of main shoots, and glandular trichomes. These results suggest that “pathomorphogenic” effects of *Hl*TFIIIA-7ZF and *Nb*TFIIIA-7ZF transgenes are similar, but not equal, despite the significant similarity on the level of encoded C2H2 zinc finger domains (Figure S5). Which protein differences are responsible for the functional differences between these two TFIIIA-7ZF variants remains to be determined.

**Figure 3:**
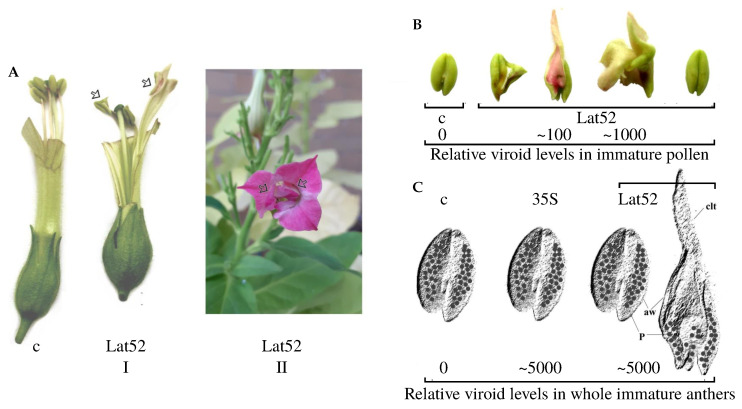
Morphogenic changes of tobacco anthers induced with elevated levels of CBCVd in immature transgenic pollen. (**A**) c: non-transformed tobacco flower with unchanged anthers; Lat52 I: flower transformed with Lat52_CBCVd_2_ containing anthers showing morphogenic changes as indicated by the arrows; Lat52 II: tobacco flower with “double corolla” containing all anthers changed to colored (pink) petal-like tissue as indicated by the arrows. (**B**) Variants of “teratomic” anther tissues. c: control anthers; Lat52: variants of anthers transformed with Lat52_CBCVd_2_. (**C**) Schematic drawing of anther and pollen variants. The numbers in (**B**,**C**) give relative, approximated levels of CBCVd (+) strands in pollen and whole immature anthers (see Figure 4). c: tissues from control plants; 35S: plants transformed with dimeric CBCVd under 35S promoter; Lat52: plants transformed with Lat52_CBCVd_2_; P: pollen; aw: anther wall; clt: corolla-like tissue.

We observed other morphological deviations mediated by CBCVd in tobacco anthers besides morphological deviations in somatic tissues caused by TFIIIA-7ZF transgenes. Surprisingly, the initiation of these changes appeared with a frequency of 20.5% (single anther change per flower) to a lower frequency of about 5% (4–5 anthers changed in the flower; see Figure 3A, Lat52, I). In very rare cases (about 0.5%), tobacco flowers developed with double corollas (Figure 3A, Lat52, II), where all immature anthers expanded to tissue-forming petal-like leaves.

**Figure 4:**
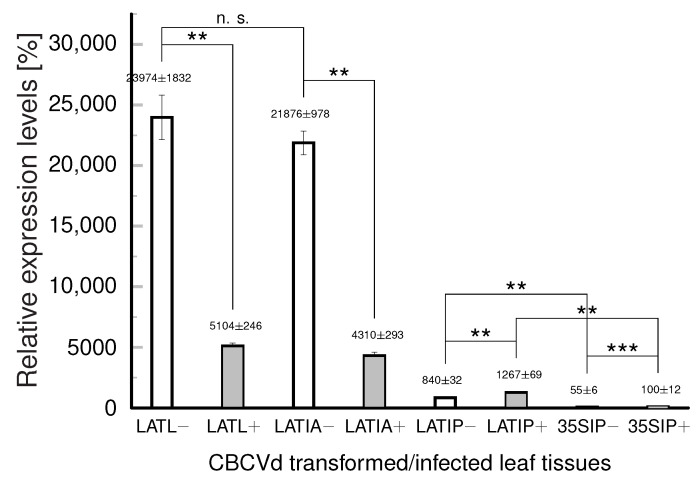
Quantification of (+) and (−) strands of viroid in anther tissues of CBCVd-transformed/ infected tobacco. Levels were compared in upper leaves (LATL), in immature anthers in the third developmental stage (LATIA), in isolated immature tobacco pollen (stage 3, LATIP; see Materials and Methods) from tobacco plants transformed with dimeric CBCVd driven by Lat52_CBCVd_2_ (see Figure S1), and in pollen collected from flowering plants transformed with dimeric CBCVd under 35S promoter (35SIP) [13]. Strand-specific qPCR was performed and the levels of CBCVd (+) strands in 35SIP were taken as 100%. Relative expression levels were normalized to 7SL RNA. Columns represent the mean ± S.D. of two replicates of each PCR reaction. Statistically evaluated differences are shown by columns connected by lines (n.s., statistically non-significant differences at *p* < 0.1; **, statistically significant differences at *p* < 0.05; ***, statistically significant differences at *p* < 0.01).

These teratomic-like changes of anthers (Figure 3B) appeared only in Lat52_CBCVd_2_ transformants. In the changed teratomic tissues, metabolic pathways have to be activated which are connected to the accumulation of phenylpropanoids and chalcones, as can be judged from levels of PAL and CHS encoding mRNAs in comparison to controls (Figure S7) and from the development of the pink coloration of corolla-like tissues (Figure 3B). However, morphogenic changes of anthers appeared neither in transgenotes driving CBCVd from 35S promoter nor in the tobacco transformed with another hop viroid, AFCVd, under Lat52 or 35S promoters (for AFCVd vectors and transgenotes see [13]). Because these results suggest that the initiation of such changes should firstly be CBCVd-specific, and, secondly, could depend on the CBCVd levels in anther or immature pollen, comparative CBCVd quantification was performed in tobacco leaf, immature anther, and pollen tissues (Figure 4). In immature anthers (stage 3, see Section 3), the level of viroid was similar to the levels in upper tobacco leaves (Figure 4). In immature pollen, the levels of CBCVd (−) and (+) strands were significantly lower than in anthers or somatic leaf tissues; however, the levels were significantly higher in immature pollen transformed with CBCVd driven by Lat52 in comparison to 35S promoter. In addition, in pollen, there was no prevalence of (−) over (+) CBCVd. We earlier explained this phenomenon through a complex process of CBCVd elimination in pollen [13,14]. The levels of CBCVd driven by Lat52 in pollen were about ten times higher than in the case of 35S as the promoter. Similarly, we found elevated viroid levels for flowering tobacco transformed with infectious Lat52_AFCVd vectors [13]. From these results, we conclude that forcing CBCVd expression in immature pollen to a higher or possibly threshold level is important to initiate “pathomorphogenic” changes in anthers, as depicted in Figure 3C.

### 2.3. Transcriptome Profiling and Identification of Some Regulatory Factors Potentially Involved in CBCVd-Caused “Pathomorphogenesis”

We showed in this and a previous study [14] that viroid-modulated changes of TFIIIA, as well as CBCVd itself, if overexpressed in pollen, can induce specific changes in somatic and generative tissues. It is expected that “pathomorphogenesis” is initiated from the misregulation of some transcription factors. To analyze the possible changes in the transcriptomes of the corresponding transgenotes in comparison to the untransformed controls, we performed transcriptome profiling based on NGS data of tobacco separately bearing *Nb*TFIIIA-7ZF in symptomatic leaf tissues and Lat52_CBCVd_2_ transgene in symptomatic anthers and compared these to normal leaf tissue and normal anthers, respectively.

In the *Nb*TFIIIA-7ZF dataset, only 215 up- and 132 down-regulated genes were detected, which satisfied a fold change |fc|≥2 with *p* < 0.05, while the Lat52_CBCVd_2_ dataset contained 12,759 up- and 6420 down-regulated genes. From the DEG comparisons we selected a set of factors showing significant expression differences and having the potential to be involved in morphogenesis networks in anthers and somatic tissues. In addition to these factors, further features can be selected from the NGS datasets. For example, a total of at least 30 different transcription factor mRNAs with no known impact on morphogenesis were significantly up- or down-regulated as well as numerous other RNAs that are involved in plant stress response (for example, heat shock proteins [20]), viroid replication (for example, RPL5 [21]), and plant immune response (for example, DICER-like, Argonaute [22,23]). A closer look at these data may improve the understanding about viroid plant-interaction and, especially, plant defense against viroid infection.

**Figure 5:**
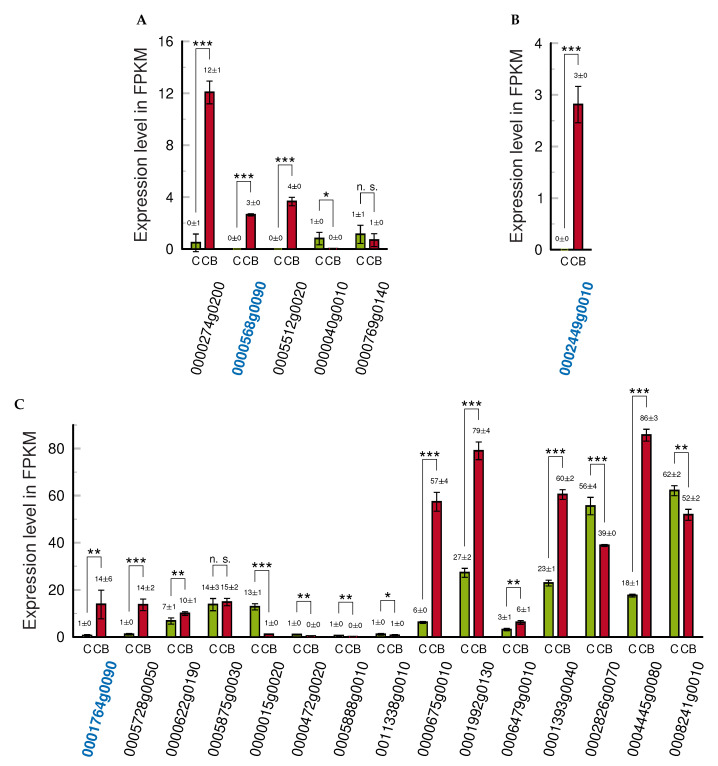
Expression of RT-qPCR candidates in CBCVd-transformed/infected anthers (red, CB) and control anthers (green, C) for (**A**) MYB family transcription factor (InterPro entry IPR015495), (**B**) MADS box transcription factor (MIXTA, IPR002487), and (**C**) ethylene-responsive transcription factors (APETALA2, IPR001471), respectively. FPKM, fragment per kilobase of transcript per million mapped reads. The x-labels are Nitab_4.5 annotations; RNAs with blue Nitab annotations were used for RT-qPCR (for example, see Figure 6). Statistically evaluated differences are shown by columns connected by lines (n.s., statistically non-significant differences at p≥0.5; *, **, ***, statistically significant differences at p<0.5, p<0.05, p<0.005, respectively.).

For petal morphogenesis, we identified MIXTA (Figure 5A), which codes for a MYB type transcription factor. It induces petal identity in the developing plant meristem [24], is functional in the morphologic development of *Antirrhinum majus* and *N. tabacum* [24,25,26], and is sufficient to induce the production of conical epidermal cells in petals [27]. A second candidate comes from the the MADS-box gene family. The ABCE model of the floral organ identity links MADS-box genes to petal development as the factor that controls the occurrence of different petal cell types [24,28], although the concrete mechanism is still unknown. A single candidate from this family was highly up-regulated in the NGS analysis and choosen as a potential candidate due to the abilities described above (Figure 5B). A third candidate occurred in KEGG maps “MAPK signaling” (map04016, Figure S3C) and “plant hormone signal transduction” (map04075, Figure S3D); both showed aberrations in ethylene-induced/-dependent pathways, linked to the number of ethylene responsive transcription factors (Figure 5C). To obtain all relevant sequences, different BLAST queries were used against the Nitab4.5 database. We used the original *A. majus* MIXTA sequence (GenBank X79108), a sequence significantly up-regulated for the etylene-responsive transcription factors (Apetala2, Nitab4.5_0000675g0010), and a sequence known from *N. tabacum* (NM_001324748) for the MADS-box gene. Resulting sequences with an identity of at least 80% with observed strong differential expressions and preferably no expression in the control state were chosen as the targets for RT-qPCR. Similarly, we identified several factors common for expression changes in anther and leaf tissues; these were mainly LITTLE ZIPPER 2-like, homeodomain basic zipper, and MADS transcription factors.

**Figure 6:**
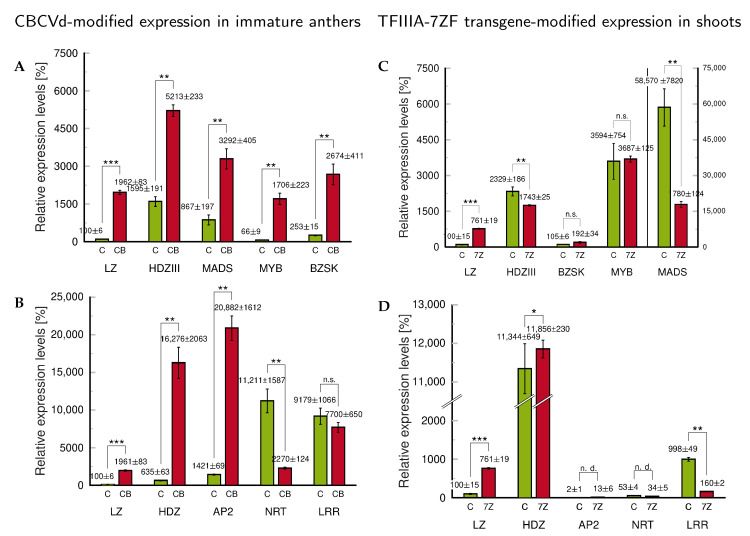
Changes in gene expression of tobacco regulatory factors (**A**,**B**) upon CBCVd-infection in immature tobacco anthers transformed with Lat52_CBCVd_2_ and (**C**,**D**) in tobacco shoots transformed with viroid-modified *Nb*TFIIIA-7ZF. The sets of factors were pre-selected from comparative transcriptomics of infected and transformed tobacco tissues. LZ: Nitab LITTLE ZIPPER 2-like, XM_016595197); HDZIII: Nitab Homeobox-leucine zipper HD-ZIPIII_Revoluta JQ686932, NM_001326077; MADS: Nitab agamous-like MADS-box protein AGL8 homolog, Nitab4.5_0002449g0010; MYB: Nitab MYB 306-like, homeodomain-like, SANT/MYB domain, Nitab4.5_0000568g0090; BZSK: Basic zipper skin Nitab4.5_0000027g0550; HDZ: Leu zipper homeobox Nitab4.5_0000241g0170; AP2: Nitab AP2/ERF domain, Apetala 2, Nitab4.5_0001764g0090; NRT: Nitab NRT1/PTR family protein 7.1-like, XM_016649644; LRR: Nitab Leucine-rich repeat receptor protein kinase MSP1-like, XM_016604323. C: controls, healthy and untransformed tobacco plants; CB: tobacco transformed with LAT52 vector bearing infectious dimeric cDNA of CBCVd; 7Z: plants transformed with plant vector bearing modified TFIIIA-7ZF cloned from *N. benthamiana*. Levels in the LZ controls were set to 100%. The mean values ± S.D. of two replicates of each PCR reaction are given. Lines connecting columns indicate statistically evaluated differences (n.s., statistically non-significant differences at *p* ≥ 0.1; *, statistically significant differences at *p* < 0.1; **, statistically significant differences at *p* < 0.05; ***, statistically significant differences at *p* < 0.01; n.d., not determined).

We used RT-qPCR analysis to compare the expression of selected factors in shoots and anthers and to confirm possible imbalanced expression; for the primers used, see Table S1. The selected set of factors included the following (for IDs see Figure 6): LITTLE ZIPPER 2-like (LITTLE ZIPPER); Homeobox-leucine zipper HD-ZIPIII (known as Revoluta in *A. thaliana*); Agamous-like MADS-box protein AGL8 homolog; MYB 306-like, homeodomain-like, SANT/MYB domain-containing protein; basic zipper skin; Leucine zipper homeobox (HDZ); AP2/ERF domain-containing protein (known as Apetala 2 in *A. thaliana*); NRT1/PTR family protein; and Leucine-rich repeat receptor protein kinase from LRR family.

For the sequences selected according to NGS analysis (MADS, MYB, and AP2), a comparison between the NGS (Figure 5) and the RT-qPCR (Figure 6) results showed that the general tendencies between both experiments were coherent. In Figure 5, sequences with a null expression in the absence of Lat52_CBCVd_2_ were deemed desirable as candidates; however, none of those sequences led to an absence of expression in the RT-qPCR. Most likely our primers were not unique to those sequences but led to the amplification of additional, closely related sequences or domains. In the NGS analysis, we found at least 15 annotated sequences for MADS, 17 for AP2, and 21 for MYB that shared at least 80% sequence identity with the candidate sequence. Any of those sequences and others, which are not annotated, may lead to the observed result. Note, we analyzed PCR products for melting points and obtained no indication of the amplification of multiple products.

To predict the possible functions of these significantly imbalanced factors we used STRING Protein-Protein Interaction Networks, Functional Enrichment Analysis (https://string-db.org/, accessed on 1 March 2023) using homologues from *A. thaliana*. These analyses pointed to the importance of analyzed transcription factors in the regulation of plant meristematic cells. Shoot apical meristems (SAMs) are tissues that function as a site of continuous organogenesis, where a small pool of pluripotent stem cells develop into lateral organs. The coordination of intercellular and intracellular networks is essential for maintaining SAM structure and size and also leads to the patterning and formation of lateral organs. Leaves initiate from the flanks of SAM and then develop into a flattened structure with variable sizes and forms. Leaf development is controlled by different components, such as hormones similar to polar auxin transport, transcription factors, miRNAs, small peptides, and epigenetic marks. Moreover, the adaxial/abaxial cell fate, lamina growth, and shape of margins are determined by certain regulatory mechanisms [29]. The SAM and lateral organs interact during plant development. Existing lateral organs influence the positions of newly formed organs to determine the phyllotaxis. The SAM not only produces lateral organs, but also influences their morphogenesis. In particular, the SAM promotes leaf polarity determination and leaf blade formation. On the other hand, lateral organs help the SAM to maintain homeostasis by restricting stem cell activity [30]. In addition, there is significant shoot morphogenetic plasticity which emerges later during post-SAM development [31], which is under intercellular communication involving a complex signaling network [32].

We detected a significant activation of the LITTLE ZIPPER 2-like gene showing an expression of 761% in the transformed tissues in comparison to 100% in the control as an imbalance due to the *Nb*TFIIIA-7ZF transgene. LITTLE ZIPPER forms a negative feedback loop with class III homeodomain-leucine zipper (HD-ZIPIII) transcription factors to confine meristematic genes specified and maintained during post-embryogenic development [33]. In *A. thaliana*, HD-ZIPIII protein (REVOLUTA) is a potent regulator of leaf polarity and vascular development. It was shown in *A. thaliana* that the overexpression of LITTLE ZIPPER proteins formed heterodimers with HD-ZIPIII, preventing it from binding to the DNA [34], and thus reducing its biological activity. In addition to the imbalance of LITTLE ZIPPER, HD-ZIPIII was significantly suppressed, reaching only 1743% in transgenic shoots bearing *Nb*TFIIIA-7ZF in comparison to 2329% in the control (Figure 6C,D). We can assume that the unusual branching of *Nb*TFIIIA-7ZF- [14] and the *Hl*TFIIIA-7ZF-transformed tobacco observed in this study (Figure 2) also involves the imbalancing of SAM resulting in isotomous dichotomy due to the growth of an equal bifurcation of the original shoot apical meristem or anisotomy [35] in case of the trifurcation of tobacco SAM [14].

**Figure 7:**
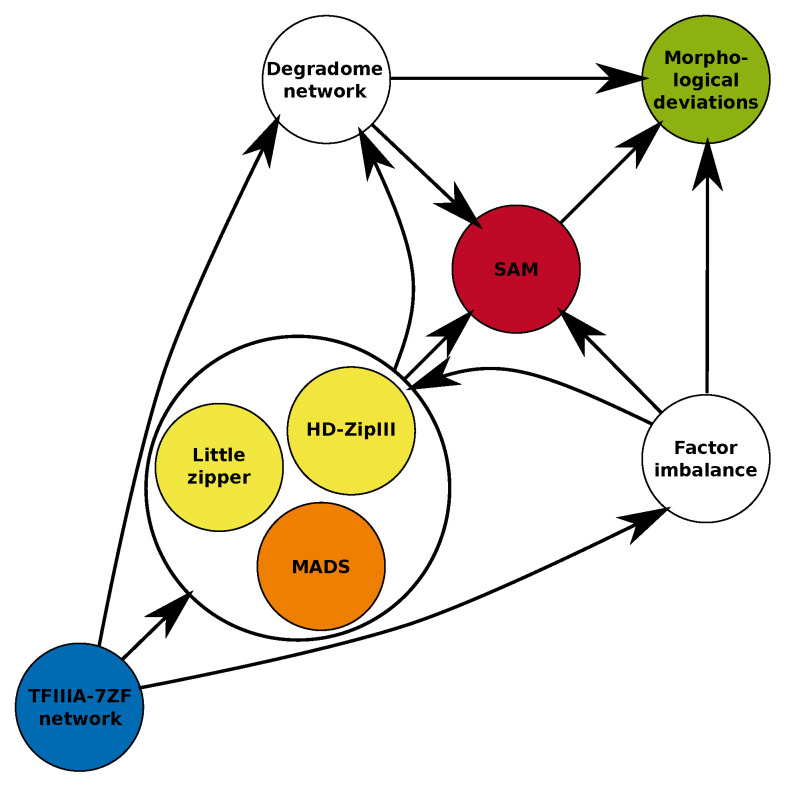
Simplified schematic drawing of potential “pathomorphogenic” changes caused due to ectopic overexpression of modified factor TFIIIA-7ZF and its pathogenic network in *N. tabacum*. Detected imbalance of expression of several factors regulating shoot and leaf morphogenesis (LITTLE ZIPPER, HD-ZipIII, MADS; see Figure 6C,D) could affect regulatory processes of shoot apical meristematic cells (SAM) and lead to unique morphological deviations observed in transgenic tobacco. Changes of downstream degradome network could influence regulation of plant development as well as viroid levels in infected plants, as documented earlier [14] and in this study (see Figure 1). The scheme is partly deduced from Protein-Protein Interaction Networks, Functional Enrichment Analysis (STRING, https://string-db.org/, accessed on 1 March 2023) using *A. thaliana* as model.

A transcription factor complex including MADS-box was significantly expressed in the upper leaves and its expression was strongly reduced in *Nb*TFIIIA-7ZF transformants from 58,569% (control) to 17,802% in transformants. MADS-box proteins have been identified to control the development and maintenance of the SAM and stem cell population in floral meristem to co-determine the development of flowers and flowering [36,37,38,39]. Moreover, MADS-box proteins can interact with homeobox, homeodomain proteins that are involved in the preservation of the spiral phyllotactic arrangement leading to a regular pattern of organ initiation, as well as in the maintenance of the stem cell fate in the shoot apical meristem, and are essential for specifying floral primordia and establishing early internode patterning events during inflorescence development [40,41,42]. Thus, an imbalance of the MADS-box network could affect the timing of flowering observed [14] and the level of K-box domain (Figure S8C) interactions and its interaction with homeobox proteins and influence developmental disorders caused in floral organs. In *Nb*TFIIIA-7ZF transformants, we previously described the splitting and the size of stigmas, shapes, and sizes of anthers and stamens [14]. It is obvious that SAM regulation is interconnected to degradome and regulation by miRNAs and ARGONAUTEs. For instance, imbalanced homeodomain-leucine zipper III (Figure S8B), which is involved in the specification of the SAM fate, is a target of miR166/165 interacting with AGO10. The deficient loading of miR166 into AGO10 results in a defective SAM [43]. The hypothetical interconnections leading from the overexpression of TFIIIA-7ZF transgenes through the imbalance of l, HD-ZIPIII, and MADS-box could influence SAM and finally be manifested as unusual abnormalities of shoots, leaves, and flowers (Figure 7). Unusual morphological deviations in tobacco were observed by other authors; for instance, the ectopic overexpression of a rice homeobox gene, OSH1, induced morphological abnormalities in the leaves, petals, and stems of tobacco transformants [44], although the character of changes was quite different in comparison to *Nb*TFIIIA-7ZF.

PCR quantification identified several factors as significantly imbalanced in *Nb*TFIIIA-7ZF tissues in comparison to the control. For example, the receptor-like serine/threonine-protein kinase homologous to At1g07650 (LRR) [45] was found to be significantly suppressed in *Nb*TFIIIA-7ZF-transformed tobacco shoots (Figure 6D). LRR is a leucine-rich repeat transmembrane protein kinase involved in transmembrane receptor protein tyrosine kinase-signaling pathway [46], plays an important role during plant growth stages, and interacts with proteins regulating procambium maintenance and polarity during vascular-tissue development and apoptosis [45,46,47]. In particular, nuclease PNI and its homologues are involved in this process as specific degradomes and play important roles in the formation of xylema tissue, the part of the plant vascular system formed by an apoptotic process [48]. In addition, PNI is involved in plant aging and senescence [49,50]. The suppression of mRNAs encoding PNI in non-senescent *Nb*TFIIIA-7ZF- and Lat52_CBCVd_2_-expressing tissues was observed previously [14] and in tobacco hybrids (Figure S6). There were other factors in shoots that we included in comparative analyses (Figure 6), which obviously were not expressed or were not differentially expressed, so one can assume no significant role in specific morphological deviations for factors from the MYB family containing homeodomain-like and SANT/MYB domains, Basic zipper skin, Leu zipper homeobox, AP2/ERF Apetala 2 homolog, and NRT1/PTR family protein (Figure 6C,D).

In case of anthers affected by Lat52_CBCVd_2_ transformation and CBCVd infection, the DEG analysis showed a very wide spectrum of changes including 12,759 up- and 6420 down-regulated genes at the given probability of *p* < 0.05. This is in accordance with the very heterogeneous “teratomic” tissues observed, which included anther-like tissues containing pollen, as well as transition stages to colored petal-like tissues (Figure 3A,B) with an enhanced expression of floral metabolome genes (Figure S7). The imbalanced expression of some morphogenesis-regulating factors in CBCVd-induced abnormalities of anthers (Figure 3) also included the up-regulation of LITTLE ZIPPER, reaching 1961% in transformed/infected tissues in comparison to 100% in the control (Figure 6A,B). In contrast to shoots, the changed anther tissues showed a significant increase in HD-ZIPIII levels from 1595% in the control (not infected anthers; Figure 6A,B) to 5212% in the changed anther tissues. The relative expression of the MADS-box within the analyzed set of factors increased from 867% (control anthers) to 3292% in the transformed/infected tissues. Some other analyzed factors potentially involved in anther “pathomorphogenesis” were changed significantly. The relative levels of the SANT/MYB factor increased from 65% in control to 1705% in transformants, Basic zipper skin increased from 253% to 2673%, and Leu zipper homeobox increase from 624% to 16,276%, while the NRT1/PTR family protein mRNA decreased significantly from 11,211% to 2269%. A non-significant decrease was observed for Leucine-rich repeat receptor protein kinase in anther tissues (Figure 6A,B). We observed an important increase in anthers for the AP2/ERF domain containing protein mRNA reaching 1421% in the control anthers and 20,882% in viroid-affected anther tissues. AP2/ERF is recognized as a plant-specific transcription factors family that contributes to plant growth, hormone-induced development, ethylene response factors, and stress [51]. AP2/ERFs are an integral component of signaling cascades including part of metabolome [52] as they regulate the expression of a wide variety of down-stream target genes related to stress response and development through different networks. This downstream regulation of transcript does not always positively or beneficially affect the plant but the plants also display some developmental defects like senescence and reduced growth under normal conditions or a sensitivity to stress conditions [53]. Basic zipper skin (bZIP42 in *A. thaliana*) is expressed in inflorescence meristem, flower, and during petal differentiation and expansion stage [54,55]. Up-regulated homeobox-leucine zipper protein from ATHB oligo family interact with other homeodomain proteins mediating plant hormone (ABA or auxin) regulatory role on growth. This homeobox zipper interacts with other HDZIP factors in the regulation of gibberellin biosynthesis and flower morphogenesis development (stamens) and is activated via the HD-ZIPIII background [56,57,58,59].

The comparison of morphogenetic changes in leaves mediated in TFIIIA-7ZF transgenotes or in *Hl*TFIIIA-7ZF × Lat52_CBCVd_2_ hybrids shows that these changes are unique and more specific (Figure 7), while “pathomorphic” anthers include much more complex and, possibly independent morphogenic and “pathomorphogenic” networks originally induced by the high level of CBCVd and/or viroid-derived small RNAs in immature pollen. Low and variable levels of these anther-deviating changes suggest an involvement of some epigenetic processes. The analysis of this level of regulation was not, however, the subject of this study and remains to be elucidated in the future.

## 3. Materials and Methods

### 3.1. Plant Cultivation Conditions, Plant Transformation, Preparation of Tobacco Hybrid Variants, and RNA Sampling

*Nicotiana tabacum* cv. Samsun plants were grown in big pots under greenhouse conditions, as described previously [14]. The isolation of immature pollen from fresh anthers or mature pollen from dehisced anthers was accomplished according to Tupý et al. [60]. Some other plant cultivations in the 2021 and 2022 seasons to perform crossings and analysis of tobacco transgenotes and hybrids for morphological differences were grown in smaller pots in climate boxes at a temperature of 25 ± 3 °C with supplementary illumination (90 μmol/m^2^/sPAR) to keep a 16 h day length.

The transformation of *N. tabacum* was performed using the *A. tumefaciens* LBA4404 bearing plant vector pJM14, constructed as described previously [13] with either *Nb*TFIIIA-7ZF or *Hl*TFIIIA-7ZF transgenes (Figure S1) cloned from *Nicotiana benthamiana* infected with potato spindle tuber viroid (PSTVd) or from *Humulus lupulus* infected with hop-latent viroid (HLVd), respectively. The transgenotes were designated correspondingly *Nb*TFIIIA-7ZF or *Hl*TFIIIA-7ZF. For the preparation of CBCVd transformed/infected tobacco lines, we used the infectious vector (CBCVd)${}_{2}$ in pFAST constructed previously [14]. This vector contained the dimeric sequence of (++) CBCVd (GenBank AC KM211547) [9] driven by pollen-specific promoter pLAT52 (Figure S2). The corresponding CBCVd-infected lines were designated Lat52_CBCVd${}_{2}$. In some comparative experiments, we used lines expressing CBCVd under 35S promoter using a vector described earlier [13]. Transformation was performed using the standard leaf disc method [61]. Regenerated, transformed plants were maintained on a medium containing 100 mg/L pf kanamycin and 200 mg/L of Timentin. Well-rooting lines on kanamycin were transferred from in vitro to the soil and subjected to further analyses. In this work, we prepared F1 hybrids between female *Nb*TFIIIA-7ZF- and *Hl*TFIIIA-7ZF-transgenic plants and Lat52_CBCVd${}_{2}$ as a source of pollen. The hybrid lines were designated as *Nb*TFIIIA-7ZF × Lat52_CBCVd${}_{2}$ and *Hl*TFIIIA-7ZF × Lat52_CBCVd${}_{2}$, respectively. In comparative experiments, seedlings from *Nb*TFIIIA-7ZF, *Hl*TFIIIA-7ZF and Lat52_CBCVd${}_{2}$, and F1 hybrids were grown simultaneously under standard conditions in climate boxes.

### 3.2. Viroid Quantification in CBCVd Transformants and Hybrids

For viroid detection, RNA was isolated from 200mg of leaves (prepared as mixed samples) with Concert™ reagent (Plant RNA Purification Reagent, Invitrogen, Carlsbad, CA, USA) according to the manufacturer’s protocol supplied for Concert reagent followed by the DNA cleavage step. For the simultaneous RT-qPCR quantification of viroid (+) and (−) strands forming thermodynamically stable structures, we used the single-strand-specific RT-qPCR method described previously [9] as a duplex reaction with housekeeping 7SL RNA, which enables the same RT conditions with *Tth* polymerase and qPCR [13,14]. This approach made the method faster and more exact. Briefly, in the first step, thermostable *Tth* polymerase was used for reverse transcription at 70 °C of viroid RNA (+) chains using CVdRTPL or (−) chains with CVdRTMI primers (Table S1). The individual primers in these first reactions were combined with an anti-beta primer (Table S1) to simultaneously generate the first strand of the 7SL RNA marker. In the second step, real-time PCR amplification was performed with the cDNAs using respective PCR FOR and PCR REV primer combinations (Table S1) for CBCVd and primer α in combination with anti-β for 7SL RNA housekeeping mRNA. The second duplex step to amplify viroid and 7SL RNA used “iQ™ SYBR® Green Super-mix” (Bio-Rad, Hercules, CA, USA) [13] with initial denaturation at 94 °C for 4 min for 40 amplification cycles (94 °C/20 s, 61 °C/40 s, 72 °C/30 s). Quantification was performed on the CFX Connect™ Real-Time PCR Detection System (Bio-Rad) with Bio-Rad CFX Maestro qPCR software v1.1. The relative viroid levels were normalized with the “delta-delta method” [62] to the levels of 7SL RNA [13,17]. For statistical analysis, we calculated the p-values for the level of significance using the two-tailed t-test, as in previous analyses [9].

### 3.3. Quantification of mRNA Levels of Selected Tobacco Genes Potentially Involved in Morphogenetic Changes and Cloning

For an analysis of the levels of mRNA 10 μg of purified and DNase was treated and the total RNA was reverse transcribed using Oligo dT18 primer and Superscript III reverse transcriptase (Invitrogen, Carlsbad, CA, USA) at 50 °C for 60 min. Reactions were performed according to [9]. For the selected genes, primers were chosen, as indicated in Table S1. The results were normalized to actin as the housekeeping control was amplified using primers NtACT, according to [13]. RT-qPCR was run on the CFX Connect™ Real-Time device (Bio-Rad, Hercules, CA, USA) as described previously [14]. Briefly, 20 μL of the reaction mixture containing 5 μL of 35-fold diluted cDNA, 5 μL of 2 μM forward and reverse gene-specific primers (Table S1), and 10 μL of 2× SYBR™ Green PCR master mix (Applied Biosystems) were run under the following amplification conditions: initial denaturation at 95 °C for 3 min, followed by 40 cycles of denaturation at 95 °C for 30 s, annealing at temperatures given in Table S1 for 30 s, and extension at 72 °C for 35 s. At the end of the reaction, the specificity of each primer pair was assessed using a melting curve analysis. The amount of a reference transcript of *Nt*actin was estimated in parallel for each sample. Ct values were measured using CFX Maestro qPCR software v.1.1 (Bio-Rad). The relative values were standardized with the delta-delta method and normalized to the sample with germinating pollen, where the calibrator was set to 100%. The data points showed the mean ± S.D. of the two replicates of each PCR reaction and the calculated statistic results are marked in individual figure legends. The sequence *Nb*TFIIIA-7ZF was cloned previously [13] from *N. benthamiana* infected with the lethal strain of PSTVd (AS1) [63]. *Hl*TFIIIA-7ZF was amplified by RT-PCR from hops (*Humulus lupulus* clone Osvald‘s 72) naturally infected with HLVd [18,64] using primers *Hl*TFIIIA7start and TFIIIAstop (Table S1). The fragment was re-amplified by *Pwo* polymerase and the blunt-end was cloned into pPCR-Script Amp SK(+) (Stratagene). Subsequently, *Xho*I, *Kpn*I restriction ends were added to *Hl*TFIIIA-7ZF using primers TFIII7Xho and TFIIIAKpn (Table S1). The cDNA fragment was then cleaved, gel-purified, and ligated to a unique site of vector pJM14 (Figure S1).

### 3.4. NGS and Transcriptome Profiling

*N. tabacum* whole transcriptome sequencing was performed in order to examine the different gene expression profiles and to find a set of genes based on gene ontology pathway information, that might be responsible for the tobacco morphogenesis. RNA isolation for NGS sequencing (Macrogen Europe Humanizing Genomics, Amsterdam, The Netherlands) was performed using the same method as described for mRNA quantification. Comparative NGS treatment and DEG analyses were performed by Macrogen and us. Reeds were trimmed with Trimmomatic v0.38 and mapped to reference genome with HISAT2 v2.1.0 [65] and Bowtie2 2.3.4.1 [66]. StringTie v2.1.3b was used for transcript assembly [67]. Differentially expressed genes (DEGs) analysis was performed using DESeq2 [68] and the R ballgown package v2.18.0 [69]. KEGG analysis was performed via the BlastKOALA webservice [70] based on the output of the expression profile for the Nitab4.5 annotated genome [71]; for an example, see Figure S3.

Sequence comparisons were carried out with DNASIS v2.6 (Hitachi Software Engineering Company, Tokyo, Japan). Protein domain sequence analyses were performed using InterProScan module of Geneious Prime® 2022.0.1 and 2023.01; the same software using option Align/Assemble-map to reference was used for NGS reads mapping to morphogenesis factor targets to pre-select analyzed factors.

## 4. Conclusions

CBCVd causes developmental distortions involving “pathomorphogenesis”, which are induced as a consequence of “forcing” its propagation by transformation in non-host and symptomless tobacco. Ectopically overexpressed viroid-modified variants of transcription factor TFIIIA, which support CBCVd replication and suppress degradome during plant development, may be part of “pathomorphogenic” networks. These CBCVd-induced networks lead to imbalanced expressions of several morphogenic transcription factors in somatic and generative tissues.

## Supplementary Materials

The supporting information can be downloaded at: https://www.mdpi.com/article/10.3390/ijms24097790/s1. References [9,13,14,15,63,72] are cited in the supplementary materials.
