# Supplementary material for: “Pathomorphogenic” Changes Caused by Citrus Bark Cracking Viroid and Transcription Factor TFIIIA-7ZF Variants Support Viroid Propagation in Tobacco
Source: Int J Mol Sci. 2023 Apr 24;24(9):7790. doi: 10.3390/ijms24097790 (PMC10178017; doi:10.3390/ijms24097790)
Supplement: Supplementary file 1 [file ijms-24-07790-s001.zip › ijms-2313443-supplementary.pdf]

Supplementary Materials: "Pathomorphogenic" Changes Caused by Citrus Bark Cracking Viroid and Transcription Factor TFIIIA-7ZF Variants Support Viroid Propagation in Tobacco

Jaroslav Matoušek [1], Kevin P. Wüsthoff [2] and Gerhard Steger [2,*]

List of Figures

S1. Schematic representation of the plant vectors bearing *N. tabacum*- and *H. lupulus*-specific TFIIIA-7ZF casettes 2
S2. Plant vector cassette used for transformation of *N. tabacum* with CBCVd infectious dimeric (++) cDNAs 3
S3. KEGG analysis of potential target genes for phenotypic changes in *N. tabacum* 4
S4. Alignment of amino acid sequences of *Nb*TFIIIA-7ZF from *N. benthamiana* and *Hl*TFIIIA-7ZF from hop 5
S5. Comparison of protein domains of TFIIIA-7ZF from *N. benthamiana* and *H. lupulus* 6
S6. Relative mRNA levels of factors forming potential viroid "degradation complex" 7
S7. Relative levels of PAL and CHS in Lat52_CBCVd$_2$ anthers 8
S8. Alignment of Zipper- and MADS-box proteins 9

List of Tables

S1. Primers 10

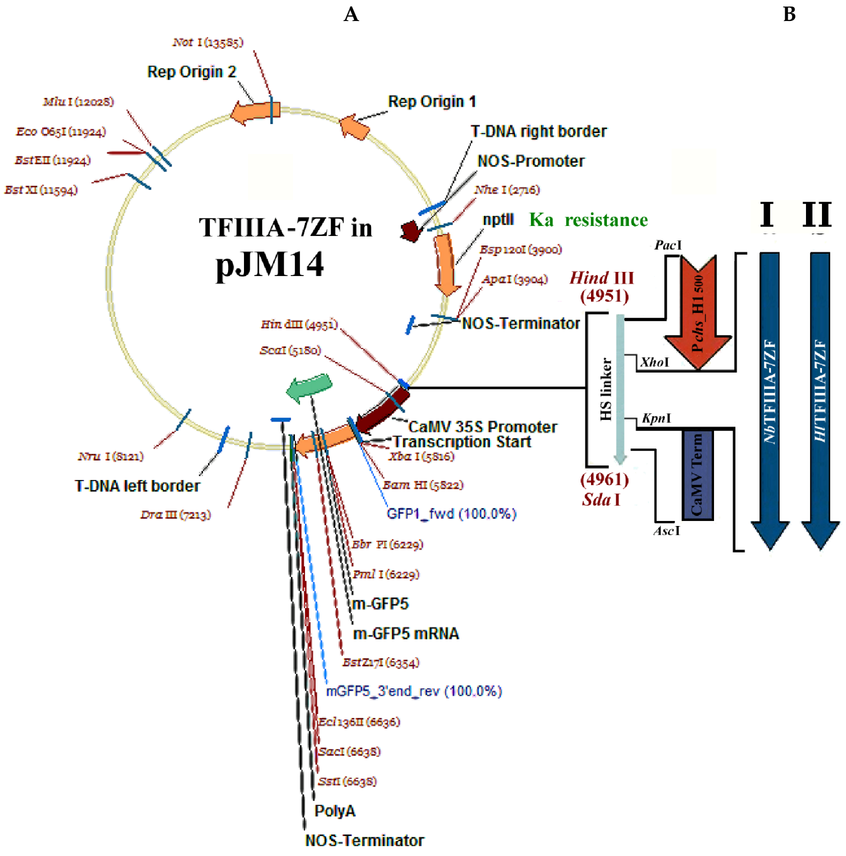

Figure S1. Schematic representation of the plant vectors bearing *N. tabacum*- (I) and *H. lupulus*- (II) specific TFIIIA-7ZF casettes driven from chalcone synthase chs_H1 promoter in pJM14. The *Nb*TFIIIA-7ZF cDNA (864 bp coding region including stop codon) and *Hl*TFIIIA-7ZF cDNA (858 bp including stop codon) were integrated, respectively in pJM14 (**A**) using the unique restriction sites *Xho*I and *Kpn*I to construct corresponding expression cassettes (**B**) [see 1, for *Nb*TFIIIA-7ZF]. The schemes in (**B**) are not to scale. The vectors provide plant resistance to Kanamycin.

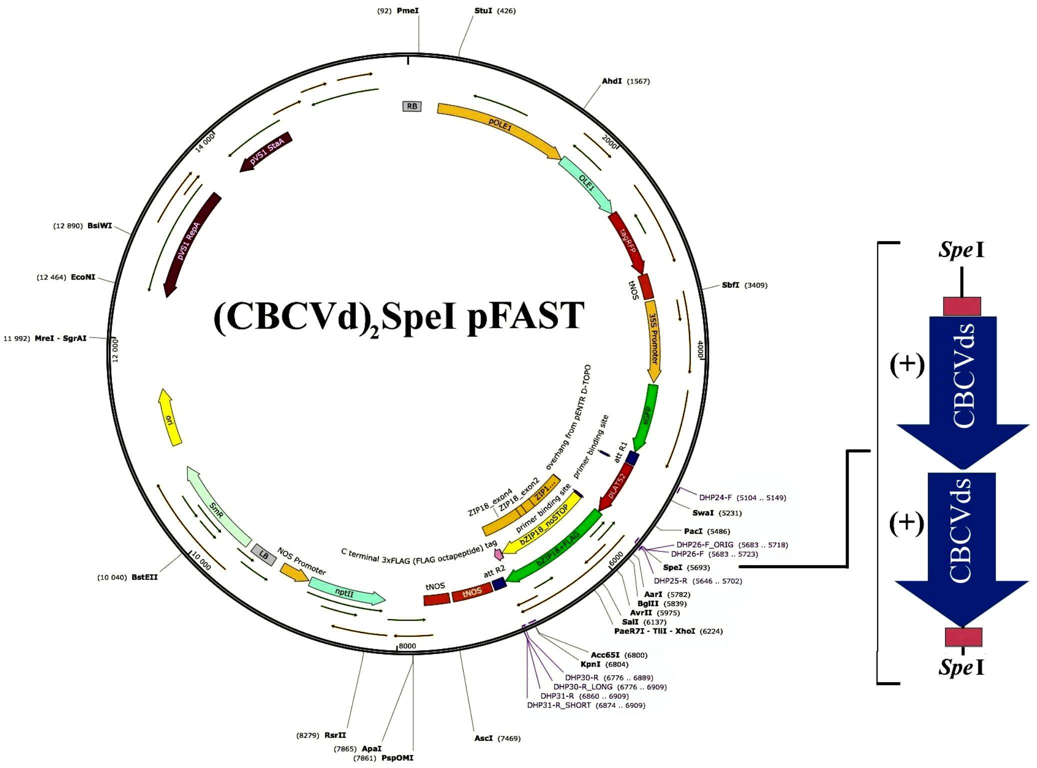

Figure S2. Plant vector cassette used for transformation of *N. tabacum* with CBCVd infectious dimeric (++) cDNAs. Plant vector pFAST bearing late pollen-specific promoter pLAT52 described previously [1] was used for transformation. Infectious (++) dimers of CBCVd were integrated into unique *SpeI* restriction site downstream of pLAT52 promoter using specific adapters [1]. The vectors were transformed to Agrobacterium LBA4404 and used for tobacco transformation. The schemes are not to scale.

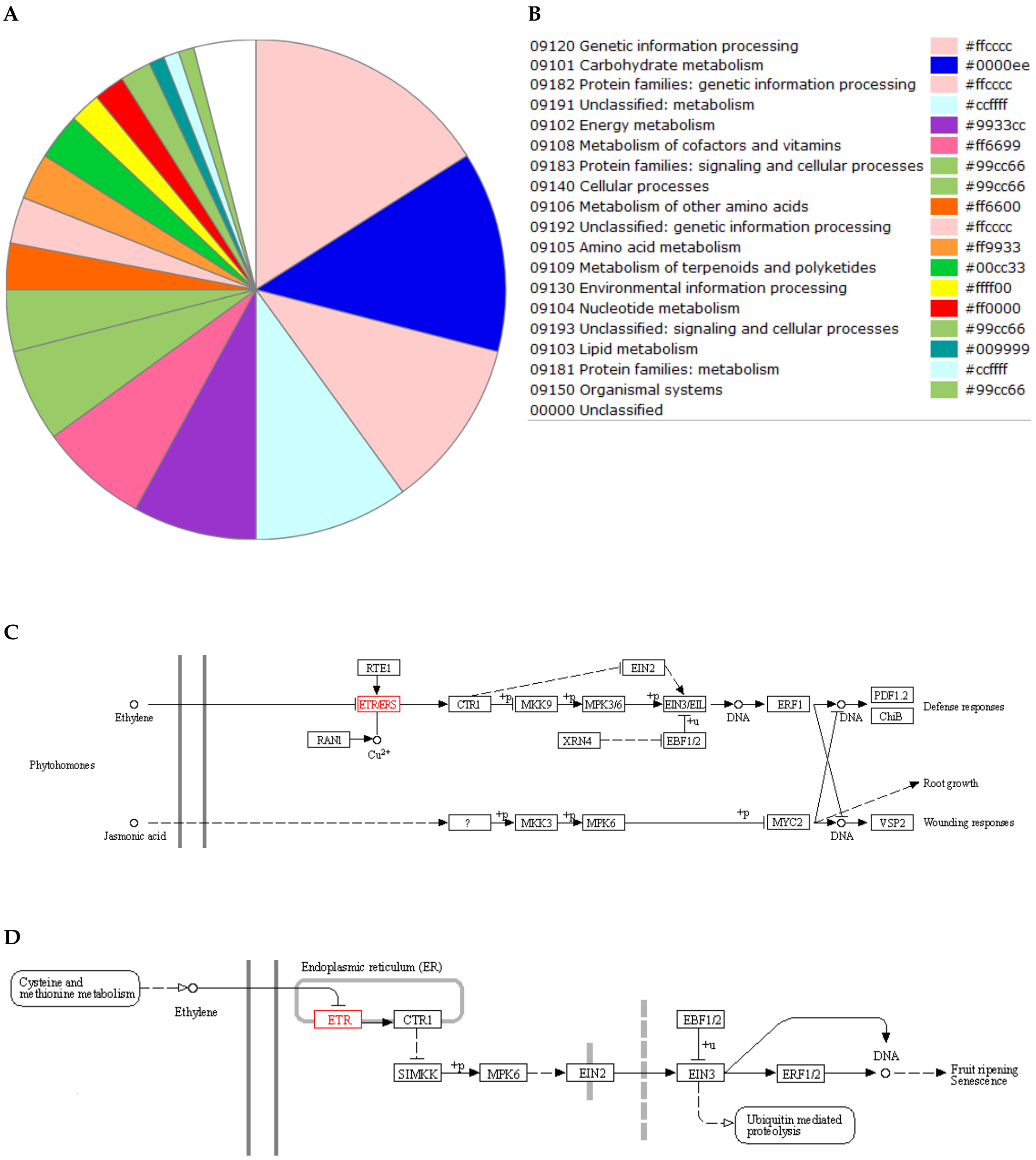

Figure S3. KEGG analysis of potential target genes for phenotypic changes in *N. tabacum*. (**A**) Distribution of 100 annotated (16.3% of 612 query entries with 4fold change and $p \leq 0.05$ from Lat52_CBCVd$_2$ dataset) protein sequences ordered after their occurrence. The most represented groups are the genetic information processing pathway with about 25% entries in 3 subgroups (16% of entries are in the biggest subgroup), the carbohydrate metabolism with about 12% in one subgroup and the signaling and cellular processing pathway with about 12% in three subgroups (6% of entries are in the biggest subgroup). (**B**) Color format used by KEGG for distinguishing between their annotated pathways. Interaction of ETR/ERS (etylene type transcription factors) inside the plants defense response (**C**) and in plant senescence and fruit ripening (**D**). As ETRs are involved in both defense response and partially in cell fate decision we took them into account as potential candidate for the phenotype change.

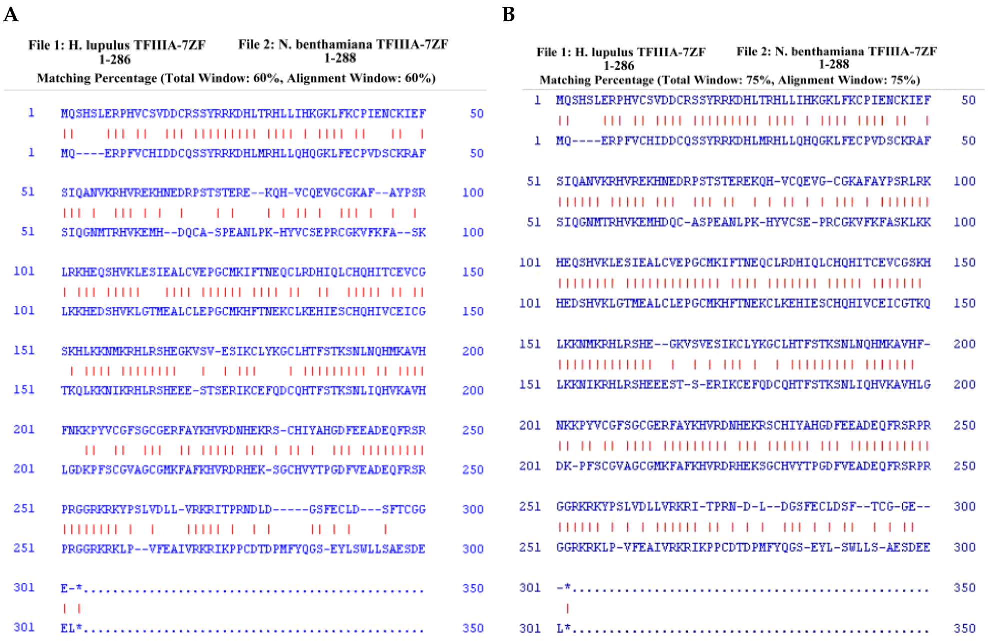

Figure S4. Alignment of amino acid sequences of *Nb*TFIIIA-7ZF from *N. benthamiana* and *Hl*TFIIIA-7ZF from hop. (**A**) aa sequences alignment using DNASIS v 2.6 with the option Compare/Maximum Matching. (**B**) aa sequences alignment using the same software, including homologous amino acids.

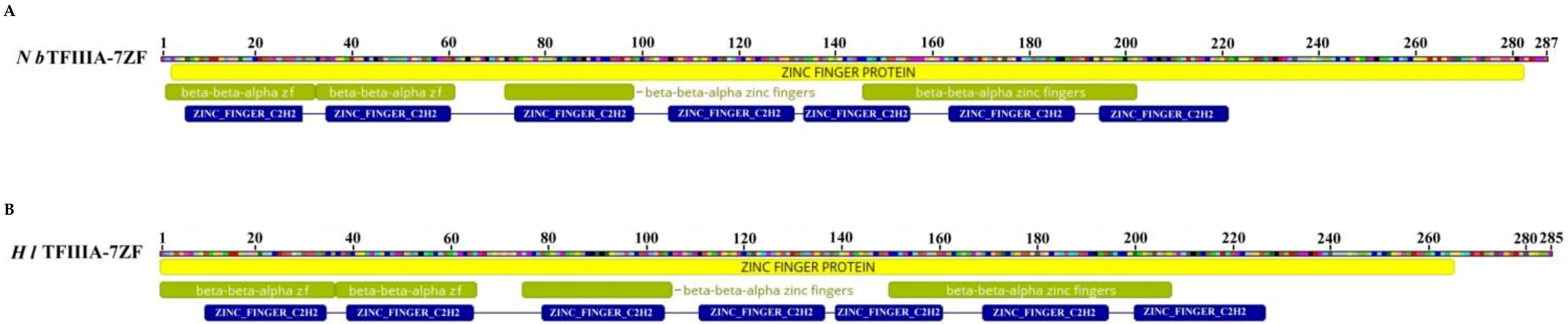

Figure S5. Comparison of protein domains of (**A**) TFIIIA-7ZF from *N. benthamiana* (*Nb*TFIIIA-7ZF) infected with lethal PSTVd strain AS1 [2] and (**B**) TFIIIA-7ZF from *H. lupulus* (*Hl*TFIIIA-7ZF) infected with hop latent viroid (HLVd) analyzed in this study. Domains were calculated and visualized using Geneious Prime 2023.0.1 software, predict option using InterProScan. Variants of TFIIIA-7ZF were cloned in vector pJM14 (Figure S1) designed earlier [1] and transformed in this study to *N. tabacum*.

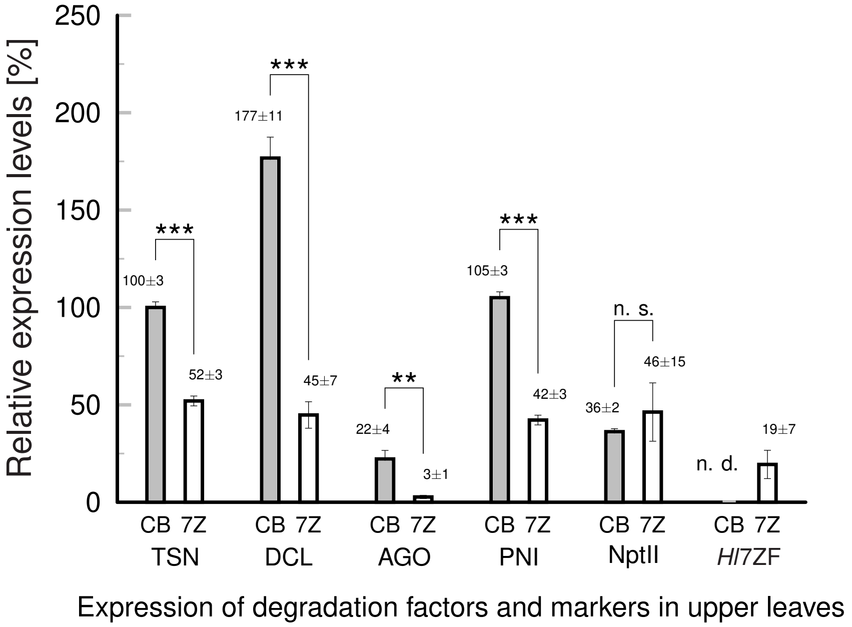

Figure S6. Relative mRNA levels of factors forming potential viroid "degradation complex" in *N. tabacum* transformed with Lat52_CBCVd$_2$ (CB) in comparison to hybrids bearing *Hl*TFIIIA-7ZF × Lat52_CBCVd$_2$ (7Z) and levels of Neomycin phosphotransferase II transgene (NptII) and hop-specific *Hl*TFIIIA-7ZF transgene in corresponding transgenic plants. Relative mRNA expression levels of tobacco factors previously analyzed in developing pollen [3] *Nt*Tudor S-like nuclease (TSN), *Nt*DCL (DCL), *Nt*AGO5 (AGO), and pollen extracellular nuclease I (PNI) were assayed using RT-qPCR in tobacco leaves collected from the upper third of plant shoots 40 days after hybrid seedlings pricking out and subsequent cultivation. RT-qPCR was performed as described in Materials and Methods were normalized to actin. The level of TSN mRNA in Lat52_CBCVd$_2$ tobacco leaves was taken as 100%. The mean values ± SD of two replicates of each PCR reaction are given. Lines with asterisks indicate statistically evaluated differences between connected values (n. s., statistically non-significant differences at $p < 0.1$; **, statistically significant differences at $p < 0.05$; ***, statistically significant differences at $p < 0.01$; n. d., not detected)

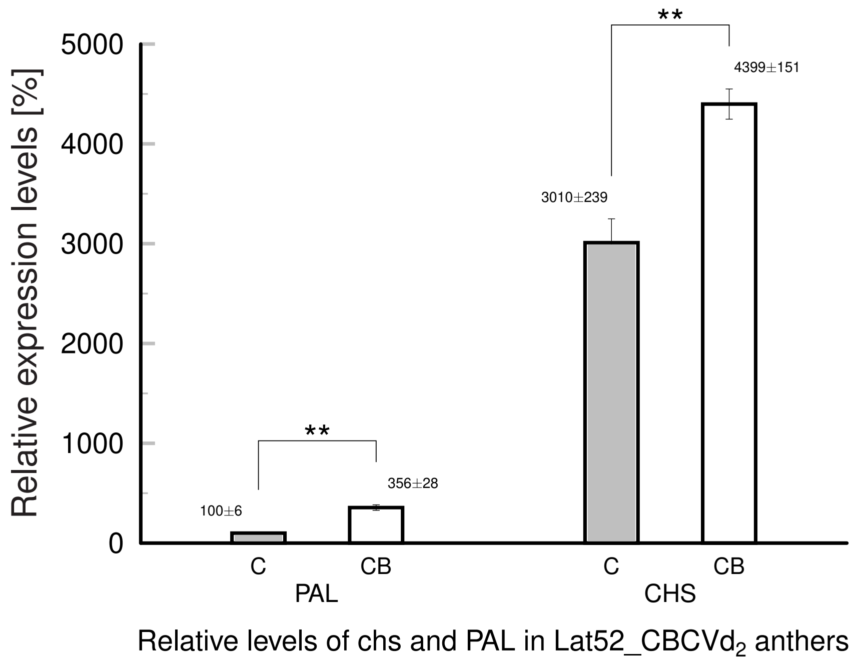

Figure S7. Relative expression levels of the metabolome genes and markers connected to coloration and morphogenic changes of CBCVd-infected tobacco anthers (Figure 4), Phenylalanine ammonia lyase (PAL) and Chalcone synthase (CHS). C, levels in non transformed control anther tissues; CB, anther tissues transformed with Lat52_CBCVd$_2$. RNA was extracted and subjected to RT qPCR analysis as described in Material and Methods. Expression levels were normalized to actin. The levels of PAL in the controls were taken as 100%. The mean values ± S.D. of two replicates of each PCR reaction is given. Lines with asterisks indicate statistically evaluated differences between selected variants (**, statistically significant differences at $p < 0.05$).

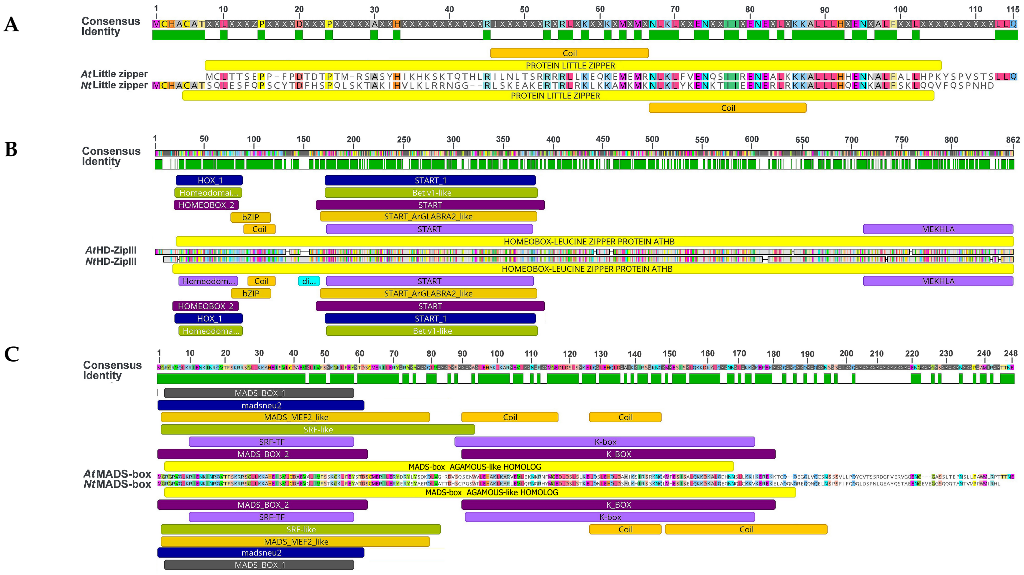

Figure S8. Alignment of amino acid sequences, domains and consensus identities of homologues of Little zipper 2 (**A**) and HD-ZipIII (Revoluta) (**B**) and MADS-box (Agamous- like) (**C**) from *A. thaliana* and *N. tabacum*. Domains designated in the figure were calculated and visualized using Geneious Prime 2023.0.1 software, Predict option using InterProScan. The domain calculations were done with set of different subprograms within InterProScan. The following platforms were used and results were presented by different colours as follows:Pfam (violet), Panther (light yellow), Coils and CDD (dark yellow), SMART (blue), PrositePatterns (dark red-violet), SuperFamily (light green), PrositeProfiles (greygreen). *N. tabacum* genomic sequences were chosen based on transcriptome profiling and blasted to protein database (https://blast.ncbi.nlm.nih.gov/Blast.cgi?PROGRAM=blastp&PAGE_TYPE=BlastSearch&BLAST_SPEC=&LINK_LOC=blasttab&LAST_PAGE=blastx) with option of organism *A. thaliana*. NCBI Acessions for the protein homologs are for *A. thalina*/*N. tabacum*: Little zipper 2-like, NP_001118868/XP_016450683; Homeobox-leucine zipper HD-ZIPIII_(*At*Revoluta) OAO94706/NP_001313006; MADS-box (Agamous-like protein AGL8 homolog) NP_568929/XP_016448958.

Table S1. Primers used for strand-specific RT-qPCR and quantification of mRNA levels by RT-qPCR.

No	Designation	Sequence 5′→3′	T/°C[a]	Purpose	Ref.
		*Hl*TFIIIA-7ZF			[4]
1	*Hl*TFIIIA7start	ATGCAAAGCCATTCTCTTGAG	56 °C	RT-PCR, cDNA cloning and RT-qPCR	
2	TFIIIAstop	TCATTCTCCTCCGCAAGTG			
3	TFIIIA7Xho	aaaCTCGAGATGCAAAGCCATTCTCTTG[b]	58 °C	cDNA cloning	
4	TFIIIA7Kpn	aaaGGTACCTCATTCTCCTCCGCAAGTG[b]			
		*Nt*ACTIN			
5	ACT-F1	TTCTGTTCCAACCATCAATGA	52 °C[c]	RT-qPCR	[5]
6	ACT-R1	GTACCACCACTGAGGACAATGT			
		7SL RNA			
7	primer-α	TGTAACCCAAGTGGGGG	61 °C	RT, RT-qPCR	[6]
8	anti-β	GCACCGGCCCGTTATCC		RT-qPCR	
		CBCVd			
9	CVdRTPL	AAGCCTGGGAGGAACAACCCAAGAG	70 °C	cDNA synthesis – reverse transcription	[5]
10	CVdRTMI	GGATCCCCGGGGAAATCTCTTCAG			
11	CVd PCR_FOR	TCACTGGCGTCCAGCACC	61 °C	RT-qPCR	[5]
12	CVd PCR_REV	AGGAAGAAGCGACGATCGG			
		degradome *Nt*AGO5			
13	1AGO5F	CAGCCTTCATCATCACAACG	55 °C	RT-qPCR	[1]
14	1AGO5R	CGTCCAACAGTTCCGTATCC			
		degradome *Nt*DICER-like homologues			
15	pollenDcl-F	GAGTGCATGAAACATATGATACAG	54 °C	RT-qPCR	[1]
16	pollenDcl-R	GAGAACTCTCAAGAAGCMTTGA			
		*Nt*PNI			
17	*Nt*PNI-F	AACGGCGACTTATCGGCACTC	53 °C	RT-qPCR	[3]
18	*Nt*PNI-R	TGGATTGCACCAGCCACACAC			
		GFP			
19	GFP-F1	CAAGAGCGCCATGCCTGAG	54 °C	RT-qPCR	[3]
20	GFP-R1	CGTGTCTTGTAGTTCCCGTCGTC			
		degradome *Nt*TUDOR S1-like nuclease (*Nt*TSN)			
21	TunucF	GTGGATGAGCCATTTGCATG	58 °C	RT-qPCR	[1]
22	TunucR	GATGCCTCAGAAGCACCAGG			
		*Nt*Little zipper			
23	LittleZ Rev	TTATGGTTTTGTTCTCTTTG	52 °C[c]	RT-qPCR	[1]
24	LittleZ For	ATCACAGTTAGAGTCATTTCAG			
		*Nt*HD-ZIPIII (Revoluta)			
25	NtRevol For	TTGAAGCTGGAACTGGTGGGAC	52 °C[c]	RT-qPCR	[1]
26	NtRevol Rev	AGCCTCAGGTGAACCAGGGAAG			
		*Nt*MADS-box			
27	Mads For	TGAAGGAGAGGGAGAAAGAGTTGG	52 °C[c]	RT-qPCR	[1]
28	Mads Rev	CCATGGTGGCATCACAGTATTAGC			
		*Nt*Myb 306-like			
29	Myb For	TGGACAGCTGAATCTCTAAG	52 °C[c]	RT-qPCR	[1]
30	Myb Rev	TATCTTCACAATTCTCACTAGC			

continued on next page

[a] Annealing temperature used in the cDNA synthesis step.
[b] Additional bases are marked by small letters; restriction sites are underlined.
[c] This annealing temperature of 52 °C was specifically derived to run samples together in one block with actin as reference.

Table S1. Continued from previous page.

No	Designation	Sequence 5′→3′	T/°C[a]	Purpose	Ref.
		*Nt*Basic zipper skin			
31	BZSK For	ACTATCTTGCATCAGCGGTAAC	52 °C[c]	RT-qPCR	[1]
32	BZSK Rev	AACTGTAAGCATCTGACGAAGG			
		*Nt*Phenylalanine ammonia lyase (PAL)			
33	NtPAL For	GCCACACATTGCCACATTCAG	60 °C	RT-qPCR	[1]
34	NtPAL Rev	GGAACGCTTCTTCAGCATTAAG			
		*Nt*Leu zipper homeobox (HDZ)			
35	HDZ For	AGAGGCTATGGAATCTGAAAGTGATG	52 °C[c]	RT-qPCR	[1]
36	HDZ Rev	CAAGAAAGCACCAGATGAAGAAATAG			
		*Nt*Chalcone synthase (CHS)			
37	NtCHS For	CTTAAGGAGAAATTTAAGCGCATG	60 °C	RT-qPCR	[1]
38	NtCHS Rev	CTGGCCCCATTCTTTGATGG			
		*Nt*AP2/ERF (Apetala2)			
39	Ap2_90 For	TGGAGTGTGGCAGAAGCGAG	52 °C[c]	RT-qPCR	[1]
40	Ap2_90 Rev	TCTGCAGCGCCATTCGTTC			
		*Nt*NRT1/PTR family protein 7.1-like			
41	NtNRT For	GGAGGATGGTATTCGGCATTTC	52 °C[c]	RT-qPCR	[1]
42	NtNRT Rev	CACCAGTCCCAGGAGGAGAATAAG			
		*Nt*Leucine-rich repeat receptor protein kinase (LRR)			
43	LRRKin For	TAGCACAATCCTTGCTGGCAC	52 °C[c]	RT-qPCR	[1]
44	LRRKin Rev	CCCTGCCATTTGTGACCATC			
		NptII			
45	NptII For	GTTGTCACTGAAGCGGGAAGG	57 °C	RT-qPCR	[1]
46	NptII Rev	GTGGTCGAATGGGCAGGTAG			

References

1. Matoušek, J.; Steinbachová, L.; Drábková, L.; Kocábek, T.; Potěšil, D.; Mishra, A.; Honys, D.; Steger, G. Elimination of viroids from tobacco pollen involves a decrease in propagation rate and an increase of the degradation processes. *Int. J. Mol. Sci.* **2020**, ***21***, 3029, [http://dx.doi.org/10.3390/ijms21083029].
2. Matoušek, J.; Kozlová, P.; Orctová, L.; Schmitz, A.; Pešina, K.; Bannach, O.; Diermann, D.; Steger, G.; Riesner, D. Accumulation of viroid-specific small RNAs and increase of nucleolytic activities linked to viroid-caused pathogenesis. *Biol. Chem.* **2007**, ***388***, 1–13, [https://doi.org/10.1515/BC.2007.001].
3. Matoušek, J.; Steger, G. The splicing variant TFIIIA-7ZF of viroid-modulated transcription factor IIIA causes physiological irregularities in transgenic tobacco and transient somatic depression of "degradome" characteristic for developing pollen. *Cells* **2022**, ***11***, 784, [https://doi.org/10.3390/cells11050784].
4. Wang, Y.; Qu, J.; Ji, S.; Wallace, A.; Wu, J.; Li, Y.; Gopalan, V.; Ding, B. A land plant-specific transcription factor directly enhances transcription of a pathogenic noncoding RNA template by DNA-dependent RNA polymerase II. *Plant Cell* **2016**, ***28***, 1094–1107, [https://doi.org/10.1105/tpc.16.00100].
5. Matoušek, J.; Siglová, K.; Jakše, J.; Radišek, S.; Brass, J.; Tsushima, T.; Guček, T.; Duraisamy, G.; Sano, T.; Steger, G. Propagation and some physiological effects of *Citrus bark cracking viroid* and *Apple fruit crinkle viroid* in multiple infected hop (*Humulus lupulus* L.). *J. Plant Physiol.* **2017**, *213*, [https://doi.org/10.1016/j.jplph.2017.02.014].
6. Matoušek, J.; Junker, V.; Vrba, L.; Schubert, J.; Patzak, J.; Steger, G. Molecular characterization and genome organization of 7 SL RNA genes from hop (*Humulus lupulus* L.). *Gene* **1999**, ***239***, 173–183, [https://doi.org/10.1016/S0378-1119(99)00352-2].
